# Copper oxide tailors multifunctional properties of fluorobarioborate glasses for optical dielectric and shielding applications

**DOI:** 10.1038/s41598-026-38663-9

**Published:** 2026-03-27

**Authors:** A. M. Abdelghany, R. M. Ramadan, M. Abdelbaky

**Affiliations:** 1 Spectroscopy Department, Physics Research Institute, National Research Centre, 33 ElBehouth St., Dokki, P.O. Box 12311, Giza, Egypt; 2 Basic Science Department, Horus University, International Coastal Road, Kafr Saad, New Damietta, Damietta, Egypt; 3 Microwave and Dielectrics Department, Physics Research Institute, National Research Centre, Dokki, Cairo, 12311 Egypt; 4 Faculty of Science, New Mansoura University, International Coastal Rd, New Mansoura City, Dakahlia, Egypt

**Keywords:** Fluorobarioborate glasses, Dielectric properties, Gamma-ray shielding, Melt-quenching technique., Chemistry, Materials science, Optics and photonics, Physics

## Abstract

The effect of CuO on the structural, optical, dielectric, and radiation shielding properties of copper fluorobarioborate (CFBB) glasses synthesized by the melt-quench technique with a general composition xCuO–(50 − x)B_2_O₃–35BaO–15MgF₂ (0–0.5 mol%) was investigated. XRD measurements indicated that all the samples, except those with higher values of CuO, were amorphous in nature. FTIR studies exhibited stable BO₃, BO₄, and B–O–B units, indicating an insignificantly modified borate network. The UV–Vis spectral studies showed enhanced UV absorption with the increase in the concentration of CuO. The value of both the direct and indirect optical band gaps is found to be almost the same (3.48–3.60 eV). Dynamic measurements of dielectric properties showed strong frequency dispersion, characterized by decreased ε′ and ε′′ and conductivity behavior based on hopping mechanisms. The radiation shielding parameters show high attenuation at low photon energies and improved shielding for CFBB glasses with increased CuO content. Hence, CFBB glasses have sufficient promise for optical transparency coupled with stable dielectric behavior and effective γ-ray attenuation. The novelty of this study is in simultaneous investigations concerning optical clarity, dielectric stability, and shielding efficacy in low CuO-doped fluorobarioborates, emphasizing their potential usage in multiple ways due to their multifunctionality.

## Introduction

 Ionizing radiation is used in a variety of sectors, including industry, scientific research, agriculture, and medicine. However, if radiation is not managed safely and with the necessary care, it could seriously harm both people and the environment during this application^1^. Shielding materials must be available to attenuate radiation in order to lessen its effects and shield personnel and other equipment from radiation’s damaging effects, thereby keeping the environment and human health safe. When choosing shielding materials, there are a few established standards, such as high density and high atomic number (Z), in addition to affordability and accessibility^2–5^.

In the past, lead was the most common material used to shield against X-rays and γ-rays. However, because of its toxicity, weight, rigidity, and poor portability, researchers have turned their attention to the field of radiation protection to discover new and alternative materials. As a result, a variety of materials, including composites and glasses, have lately been investigated through computer modeling or experimentation as potential superior substitutes for conventional materials^6–8^. However, there are still a lot of unanswered questions, and additional research in these areas is needed. Thus, the goal of this effort is to use glass to create a different, eco-friendly material for gamma-radiation protective applications^9–12^.

Bariumborate-based glass systems with transition metal oxides have been the subject of extensive investigation, mainly due to their attractive prospective uses in radiation shielding, optical devices, and other technological fields. By adjusting the glass’s chemical composition and dopant concentrations, these glasses can be tailored to exhibit beneficial optical and radiation shielding capabilities. A class of heavy-metal-oxide glass blocks known as lead barium borate is based on a combination of PbO, BaO, and B_2_O_3_ elements^13^. The well-known characteristics of pure borate glasses include their great sensitivity to active dopants, broad transparency window, and low phonon energies^14^. Lead and barium-containing heavy-metal-oxide glass significantly improves the densities and refractive indices of the host glass while preserving high optical transparency^15^. They can now be used in specialized optical devices as a result. Moreover, the elevated levels of Pb and Ba provide efficient shielding against high-energy gamma radiation^16^. By altering the respective ratios of the three components, these systems’ physico-optical and radiation attenuation properties may be changed^17,18^.

Binary alkali borate glasses have long been of interest to scientists due to their remarkable differences in physical features and structural composition, which provide a varied range of chemistry. The phrase “borate anomaly” refers to a variety of these physical features. As the quantity of alkali oxide rises, there is an odd observation that the direction of thermal expansion is changing^19,20^.

 In view of the greatest 4-coordination observed at 35–40 mol% alkali oxide and the fluctuation in the thermal expansion coefficient at 16–20 mol%, the earlier theory linking the anomaly to the development of nonbridging oxygens was reexamined^21^. The origin of this behavior has been suggested to be the development of big, awkward tetraborate groups^22^. Anionic conduction in borate glass is enhanced by the substitution of fluoride ions for oxygen ions. With the optical qualities of a fluoride crystal and the physical attributes of an oxide glass combination, the glass-ceramic material “blended oxyfluoride borate” is an intriguing one. Because of this, it is a special kind of hybrid optical material that combines the benefits of crystal and glass structures^23^.

As the function of the fluoride (F^−^) ion is still unclear, further research is required to clarify and comprehend the many characteristics of fluoridated glasses that are similar to oxide glasses in composition. Glasses containing three-dimensional transition metal ions are ideal for a variety of specialized applications due to their distinct optical, electrical, and magnetic characteristics^24–27^.

The various physical features of these materials are thought to be caused by the capacity of three-dimensional transition metal (TM) ions to exist in several coordination or valence states. The specific coordination and valence environment that the TM ions adopt are mostly determined by elements like the kind of glass system, melting temperature, and composition of the glass. These glasses have tunable physical properties because the TM dopants have numerous stages of accessibility for oxidation and coordination. The primary goal of this work is to describe the produced glasses sample containing the fundamental ingredients of xCuO-(50-x)B_2_O_3_−35BaO-15MgF_2_ by combining characteristics, including optical, density, and shielding properties. These investigations should shed some light on how the measured collective properties of glasses with fluoride and oxide that include CuO differ from one another^28–30^.

Because of its special optical, structural, and radiation-attenuation qualities, copper fluorobarioborate (CFBB) glasses are a cutting-edge family of materials with uses in radiation shielding, structural engineering, and optics. These qualities support a range of applications in the following ways. First of them, optical Applications which include UV and IR Transparency: CFBB glasses can be made to exhibit high transmittance in ultraviolet (UV) and infrared (IR) regions, which makes them valuable for optical windows and lenses in scientific and industrial instruments, Nonlinear Optics: The presence of Cu^2+^ ions in CFBB glasses enhances their nonlinear optical properties, making them useful for applications such as laser technology, optical switching, and photonic devices and Luminescence and Photonics: these glasses exhibit good fluorescence characteristics, making them useful in optical amplifiers, LEDs, and fiber optics). The second of them, Structural Applications: The mix of borate, fluoride, and barium oxides improves the glass’s mechanical and thermal durability, making it appropriate for high-stress applications.

Structural Reinforcement: By adding these glasses to coatings and composites, the stability and longevity of optical and electronic components can be improved. Third of them, Radiation Shielding Applications that include, Gamma-Ray Shielding: CFBB glasses have significant gamma-ray attenuation, which makes them appropriate for use in radiation protection windows and nuclear facility shielding. This is especially true when heavy elements like barium (Ba) and copper (Cu) are included. Neutron Shielding: These glasses can be used in nuclear reactors and for medical radiation shielding because of their boron concentration, which aids in neutron absorption.

X-ray Protection: These glasses’ high density makes it possible for them to efficiently reduce X-ray radiation, which is advantageous for diagnostic and imaging settings in medicine.

From a biophysical point of view, the very basics of research into radiation shielding materials concern the interaction of ionizing radiation with biological tissues and matter. Gamma rays and X-rays may deeply penetrate an organism and ionize water molecules and biomolecules, leading to the generation of free radicals that can damage DNA, cell membranes, and proteins. For example, in medical radiology or nuclear medicine facilities, healthcare workers are at risk of cellular mutations or necrosis due to chronic exposure to γ-radiation in cases of poor shielding.

Materials with high atomic numbers and densities, such as Ba- and Pb-based glasses, are effective because they enhance photoelectric absorption and Compton scattering, thereby reducing the intensity of radiation that reaches biological tissues. For example, a BaO–B₂O₃ glass window in a radiotherapy room can significantly reduce γ-ray penetration, minimizing patient and operator exposure. The introduction of CuO and MgF₂ into the glass matrix further optimizes optical transparency and radiation attenuation without toxicity unlike lead, which poses severe biological risks.

The proposed CuO–BaO–B₂O₃–MgF₂ glass materials serve as an environmentally friendly biophysical shield, effectively safeguarding human health by reducing the biological impact of ionizing radiation. The current research aims to meet the urgent demand for lead-free, functional glasses that possess the ability to simultaneously protect from ionizing radiation and exhibit optical and dielectric functionalities by studying the remnant potential of the newly discovered copper fluorobarioborate (CFBB) glass family, but this research specifically aims to explore the potential of the CFBB glass system, xCuO-(50 − x)B₂O₃−35BaO-15MgF₂ (0 ≤ x ≤ 0.5 mol%), which would arise from the specific minimal doping amounts of the optically/electrically active ion, Cu²⁺, to potentially provide potential functionalities without impeding the glass structure in an attempt to provide a new and advanced functional material system, as emphasized by the current research and emphasized by the need for new research on the topic of lead-based glass systems^31–34^.

## Materials and methods

### Preparation

The glass compositions *x*CuO-(50-*x*)B_2_O_3_−35BaO-15MgF_2_ (0 ≤ x ≤ 0.5 mol%) were prepared employed high-purity laboratory materials. Boron oxide was derived from orthoboric acid (H_3_BO_3_), while barium and magnesium oxide were introduced via barium carbonate (BaCO_3_) and magnesium fluoride (MgF_2_). The addition of copper was in the form of copper oxide (CuO). Glass preparation was carried out in an electric furnace under standard atmospheric conditions. The starting materials were combined in porcelain crucibles, and the melting process was conducted at temperatures ranging from 1050 to 1200 °C, depending on composition, for 30 min. The melt was swirled frequently and then poured onto a stainless steel plate and pressed by another plate to get a disc-like shape. All samples were homogeneous and transparent, and the samples were kept in a dissector until required. Detailed compositions of the investigated glasses are available in Table 1.

### Sample characterizations

Structural analysis was conducted via X-ray diffraction using a Bruker D8 Advance system with Cu Kα radiation source. The measurements, performed on powdered samples, covered a diffraction angle (2θ) from 4° to 70° with 0.4 s intervals to determine the amorphous or crystalline character of the materials.

The glass samples were analyzed using FTIR spectroscopy at room temperature, employing the KBr disk technique. Measurements were performed on a Nicolet is10 FTIR spectrometer with a resolution of 2 cm⁻¹ across the 400–4000 cm⁻¹ wavenumber range. Following spectral correction, quantitative analysis was conducted using deconvolution methods.

Optical characterization was performed using a double-beam spectrophotometer (JASCO 570, Japan) on polished glass samples of approximately 2 mm thickness. The UV-visible absorption spectra were recorded at a wavelength range from 190 to 1000 nm, with 0.3 nm accuracy. The spectra were used to evaluate the optical band gaps, refractive indices, and optical primitivity of each glass sample.

## Results and discussion

*x*CuO-(50-*x*)B_2_O_3_−35BaO-15MgF_2_.

**Table 1:** Values of N_4_ for all CuO concentrations.

Sample Code	CuO	B_2_O_3_	BaO	MgF_2_	*N*_4_	D (g/cm^3^)	V_m_ (cm^3^/mol)
BaBMgF-Cu0	0.0	50.0	35.0	15.0	0.598	3.521	27.783
BaBMgF-Cu1	0.1	49.9	35.0	15.0	0.587	3.524	27.757
BaBMgF-Cu2	0.2	49.8	35.0	15.0	0.592	3.530	27.717
BaBMgF-Cu3	0.3	49.7	35.0	15.0	0.573	3.548	27.581
BaBMgF-Cu4	0.4	49.6	35.0	15.0	0.591	3.548	27.584
BaBMgF-Cu5	0.5	49.5	35.0	15.0	0.589	3.548	27.586

### X-ray diffraction

**Fig. 1:**
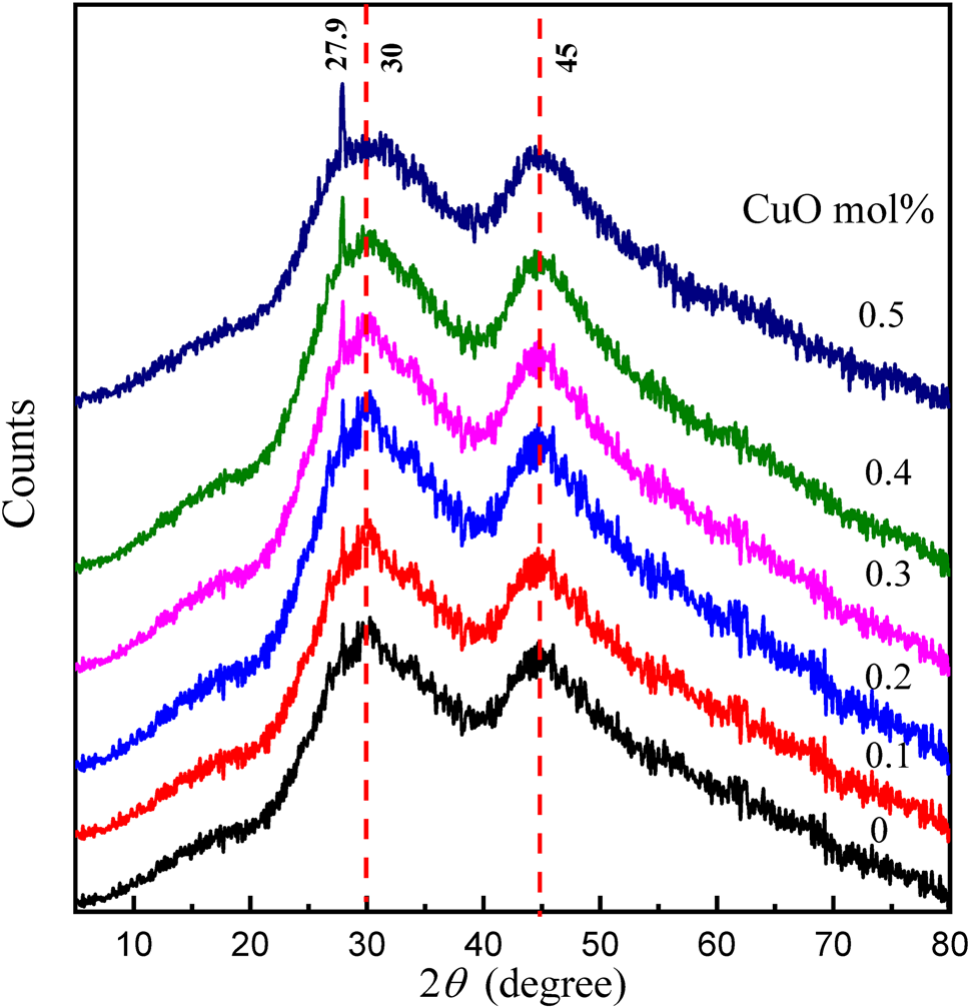
XRD pattern of the *x*CuO-(50-*x*)B_2_O_3_−35BaO-15MgF_2_ Glasses.

Figure 1 shows the X-ray diffraction (XRD) pattern of the studied borate glass, which shows two broad bands due to the dual role of BaO and MgF_2_ centered at 30° and 45°, which is characteristic of an amorphous borate network. These broad bands indicate the presence of short-range order in the glass structure with a lack of long-range periodicity. The two broad bands indicate that the matrix has two different amorphous phases. At 2*θ * = 30^o^ refer to the amorphous state of the main borate matrix. The main amorphous borate network is typically credited with this band. The backbone of these glasses is made up of the borate matrix, whose disordered structure causes a diffuse diffraction maximum at around 30°. In glasses made of borate, while long-range crystallinity is absent, short-range order is frequently preserved, and 2*θ * = 45 °, this band may be related to the BaO and/or MgF_2_ in this range of composition. The presence of BaO and/or MgF_2_ in the glass is associated with the second diffuse characteristic that was seen at about 45°. Localized areas with a marginally varied short-range ordering may result from these factors. These areas are still amorphous, but an extra-wide diffraction peak is produced by their unique structural surroundings. This behavior points to a heterogeneous distribution of the modifiers inside the borate matrix or a type of phase separation.

Optical and Structural Performance: The glass’s optical clarity and physical stability may be impacted by the existence of two amorphous phases. While the secondary phase involving BaO and/or MgF_2_ may affect elements like refractive index or thermal stability, the borate phase usually controls the overall optical characteristics. Customizing Compositions: When maximizing the glass composition for particular uses, it is essential to comprehend these diffraction characteristics. It is possible to get the required levels of optical transmission, mechanical durability, or even radiation shielding capabilities, for example, by adjusting the balance between the borate network and the BaO/MgF₂-rich areas.

However, with the addition of a relatively higher content of copper oxide replacing boron oxide, a small, sharp peak appears in the XRD pattern centered at around 27.9°. This sharp peak suggests the formation of a crystalline phase within the amorphous glass matrix. This may be interpreted in terms of the formation of copper oxide crystallites, such as CuO or Cu_2_O, within the glass matrix, or phase separation resulting from copper-rich regions or clusters. Different authors attribute the formation of such a peak to the structural rearrangements within the glassy matrix, giving rise to the presence of mixed amorphous-crystalline character, with the crystalline phase emerging due to the higher copper content replacing boron oxide.

From the structural study, it has been clearly made out how this research has one particular vastly important novelty: the Cu²⁺ ion concentration in the CFBB structure up to 0.3 mol% can be optimized as a non-toxic modifier without destroying the borate structure in the amorphous construct. On the contrary, 0.5 mol% is a limit of solubility. Such a specific determination of the limit of dopant accommodation is a decisive moment of the design of the amorphous structure in this particular CuO-fluoroborate.

### FTIR absorption spectra

**Fig. 2:**
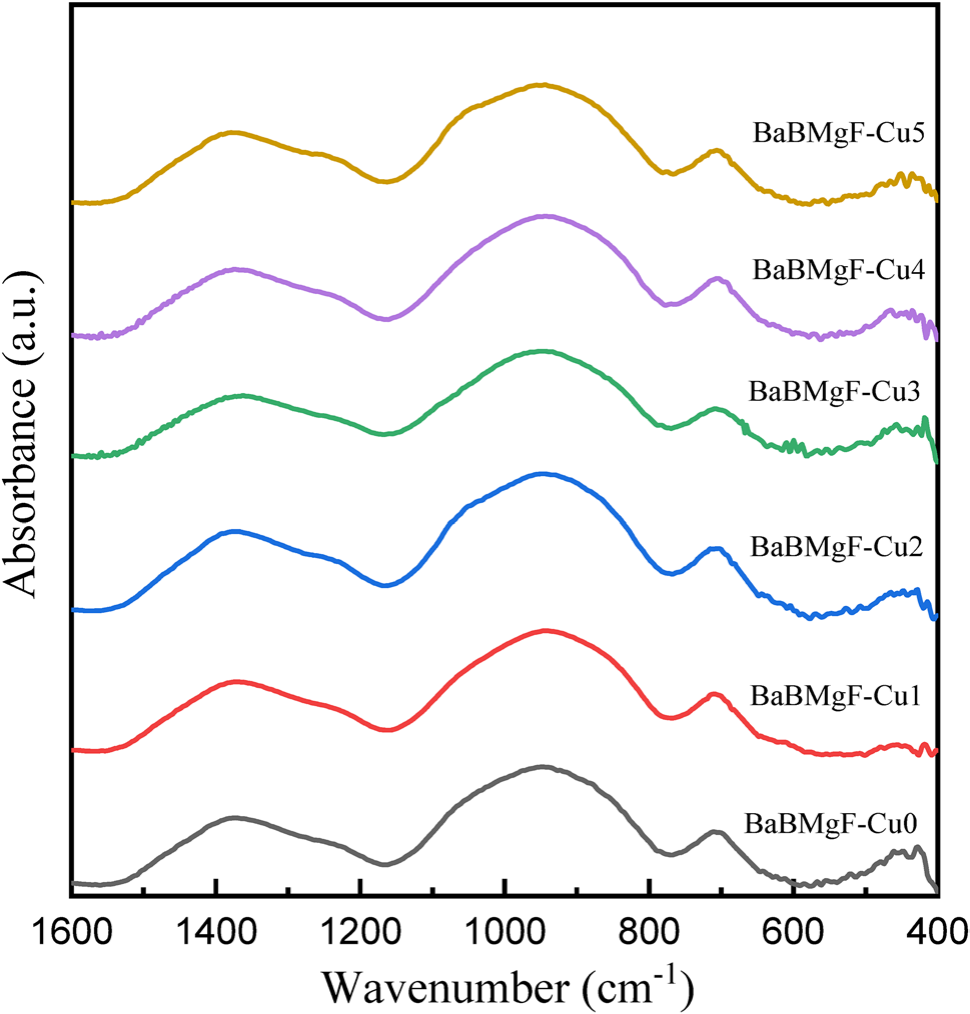
Normalized FTIR spectra of the *x*CuO-(50-*x*)B_2_O_3_−35BaO-15MgF_2_ Glasses.

Figure 2 displays the normalized FTIR spectra of the *x*CuO-(50-*x*)B_2_O_3_−35BaO-15MgF_2_ glasses under examination. From 400 to 4000 cm^− 1^, the prepared samples’ FTIR bands extend. Qualitative interpretations are the only information that can be derived from this figure. The spectra exhibited two dominant absorption regions centered at 1000 cm^− 1^ and 1300 cm^− 1^. Certain borate groups, such as the diborate, triborate, tetraborate, and pentaborate groups, produce the majority of the absorption bands in the borate glasses due to the B-O stretching vibration of the tetrahedral [BO_4_]. The B-O band [BO_3_] units in borate groups with bridging oxygen are responsible for the other regions, which range from 800 to 1200 cm^− 1^. In addition to an absorption band at 700 cm^− 1^ in all glass spectra is attributed to the bending vibration of B–O–B in symmetric BO_3_ triangle groups. Additional examples are orthoborate, pyroborate groups, and metaborate chains and rings that do not produce non-bridging oxygens (NBOs)^35^. Moreover, Low wavenumber area (< 500 cm^− 1^) absorption is thought to be caused by vibrations of the cations Ba^+ 2^, Mg^+ 2^, F^−^and Cu^+ 2^ in their network sites. With the increase in the content of Cu in the prepared sample, it is found that there is no noticeable differentiation in the FTIR Spectrum.

**Fig. 3:**
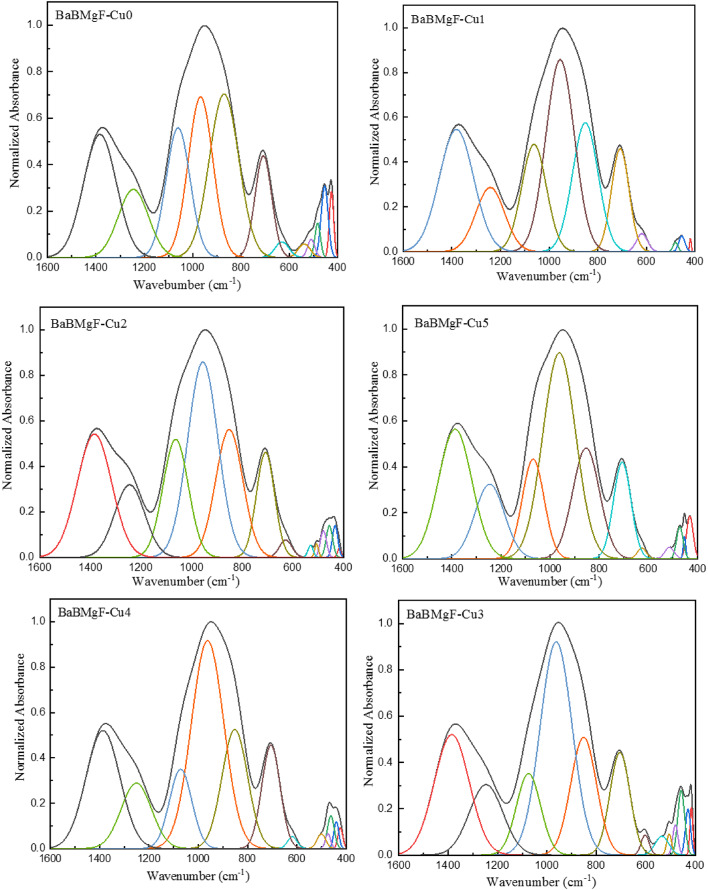
Deconvolution FTIR spectra of the *x*CuO-(50-*x*)B_2_O_3_−35BaO-15MgF_2_ Glasses.

The structural groups within the studied glasses were quantitatively analyzed through deconvolution of the IR spectra [15], which provides detailed explanations of the deconvolution process and band analysis. Deconvolution of the spectra shown in Fig. 3 enabled the determination of the four-coordinated boron fraction (*N*_4_) in these glasses. This methodological approach has demonstrated reliability in calculating *N*_4_ values for borate-based system^36^.

To understand the role of BaO with MgF_2_ in the borate glass structure. Requires examination of structural differences between binary BaO-B_2_O_3_^37^ and BaF_2_-B_2_O_3_^38^ glass systems. In BaO-B_2_O_3_ glasses, BaO converts planar BO3 groups to BO4 tetrahedra in various formations without creating non-bridging oxygens (NBOs). The fraction of four-coordinated boron (*N*_4_) increases with BaO content up to 35 mol%, after which it decreases due to BO_4_ breakdown into three-coordinated boron with NBOs. Conversely, in BaF_2_-B_2_O_3_ glasses, BaF_2_ partially modifies the borate matrix through F^−^ ions, converting BO_3_ to B_4_ in the form of the $Ba_{{1 \mathord{\left/ {\vphantom {1 2}} \right. \kern-\nulldelimiterspace} 2}}^{2 + }{[B{O_3}F]^ - }$units connected via B-F.Ba^+ 2^.F-B linkages. The remaining BaF_2_ forms BaF_4_ tetrahedra in either amorphous or crystalline phase^38^.

The studied glasses exhibit two different four-coordinated borate configurations. These configurations arise from two distinct modification processes: first, when BaO interacts with the borate network to form BO_4_ units, and second, when MgF_2_ interacts, leading to the formation of B_4_ as formula$Mg_{{1 \mathord{\left/ {\vphantom {1 2}} \right. \kern-\nulldelimiterspace} 2}}^{2 + }{[B{O_3}F]^ - }$ units. The percentage of four-coordinated boron atoms (***B***_4_) is determined by calculating the proportion of four-coordinated units (*A*_4_) relative to the sum of all coordinated units (*A*_4_ + *A*_3_), where *A*_4_ encompasses both BO_4_ and B_4_ units, and *A*_3_ represents the BO_3_ units.

The peakfit tool was used to assess the function groups’ overlapping bands attached to the glass matrix during the deconvolution process. The deconvoluted FTIR spectra of the samples, CuO 0, 0.1, 0.2, 0.3, 0.4, and 0.5, are displayed in Fig. 3. The following formula was used to determine the quadruple groups based on the deconvolution data that was acquired^39^.

1 $$\:{N}_{4}=\frac{{A}_{4}}{{A}_{4}+{A}_{3}}$$

**Fig. 4:**
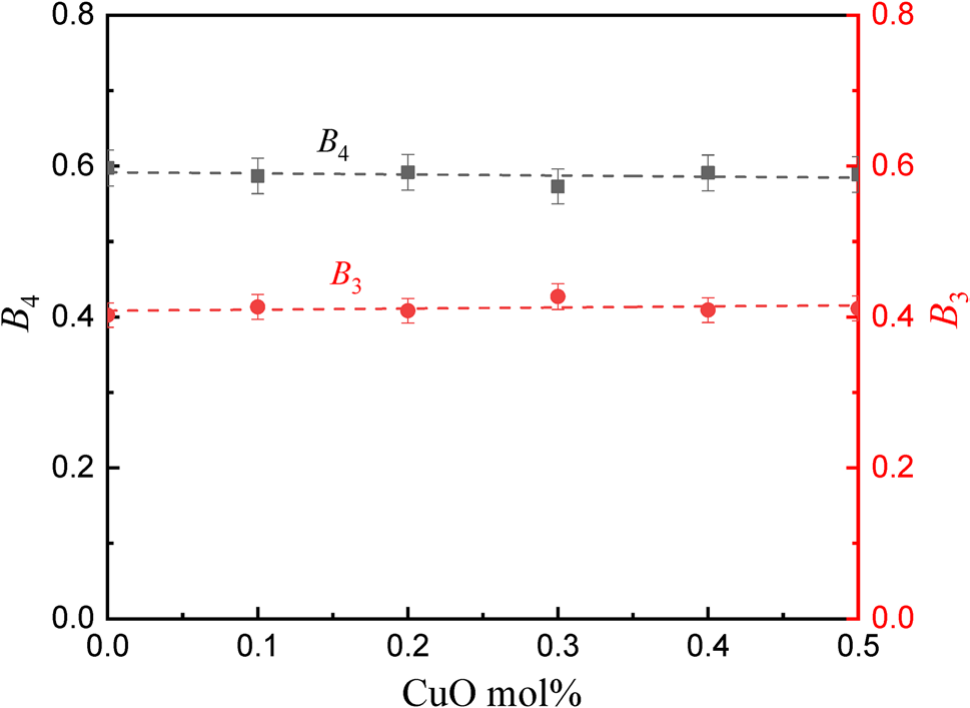
Fraction of B_4_ and B_3_ as a function of CuO content.

Figure 4 illustrates how B_4_ and B_3_ behaved when CuO content was added. It was found that there are hardly any changes in the values of B_4_ and B_3_. In addition, the values of (N_4_) in Table 1 prove the results.

### Density and molar volume

**Fig. 5:**
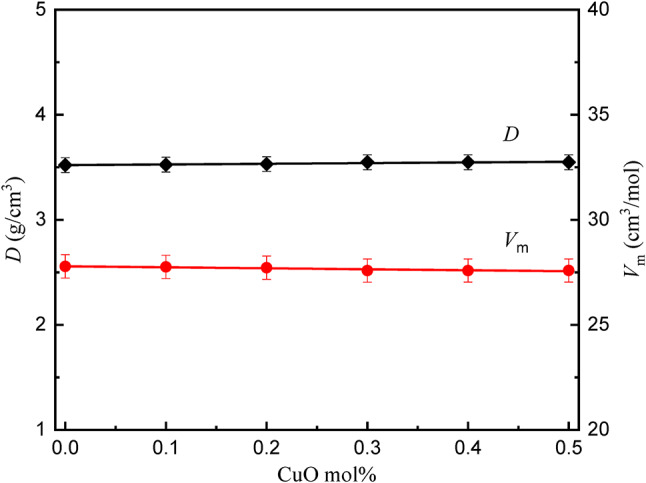
Density and molar volume of the *x*CuO-(50-*x*)B_2_O_3_−35BaO-15MgF_2_ Glasses.

**Fig. 6:**
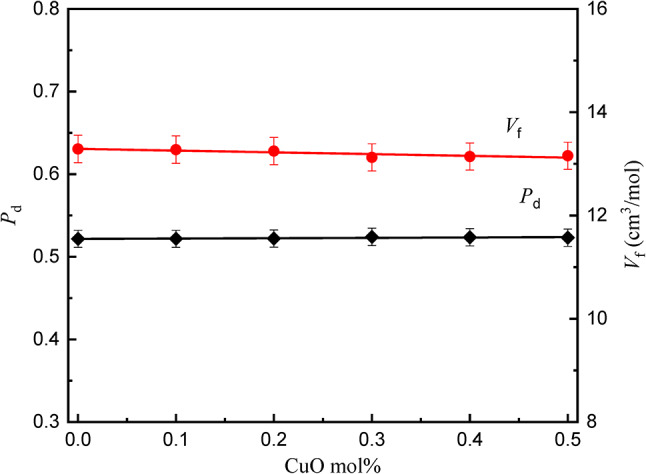
Pacing density and free volume of the *x*CuO-(50-*x*)B_2_O_3_−35BaO-15MgF_2_ Glasses.

Density and molar volume of the glass compositions are given in Table 1. Figure 5 represents the relation between density (*D*) and molar volume (*V*_m_) for the studied glasses. Both density (D) and molar volume (*V*_m_) exhibit negligible changes when B_2_O_3_ is replaced by CuO, since the overall molecular mass of the glasses only varies slightly from 9781.9 to 9786.87 with changing CuO content, and it can be considered effectively constant. However, the interdependence of these properties is intrinsically linked to the structural configuration of the glass matrix. Moreover, both free volume (*V*_f_) and packing density (*P*d) remain nearly constant, as the structure of the studied glass remains constant, as shown in Fig. 6.

The current findings of a “matrix saturation” effect, the plateau in molar volume for CuO ≥ 0.3 mol%, also present new physical evidence of structural accommodations being approached. This suggests CuO doping grows more restrictive in filling free volume, but after a threshold, it contributes to ion clustering, which occurs before crystal formation, as seen by XRD. This refinement of density/molar volume correlation enables predictive optimization of doping levels before structural instability takes place.

### Optical absorption for the studied glass samples

**Fig. 7:**
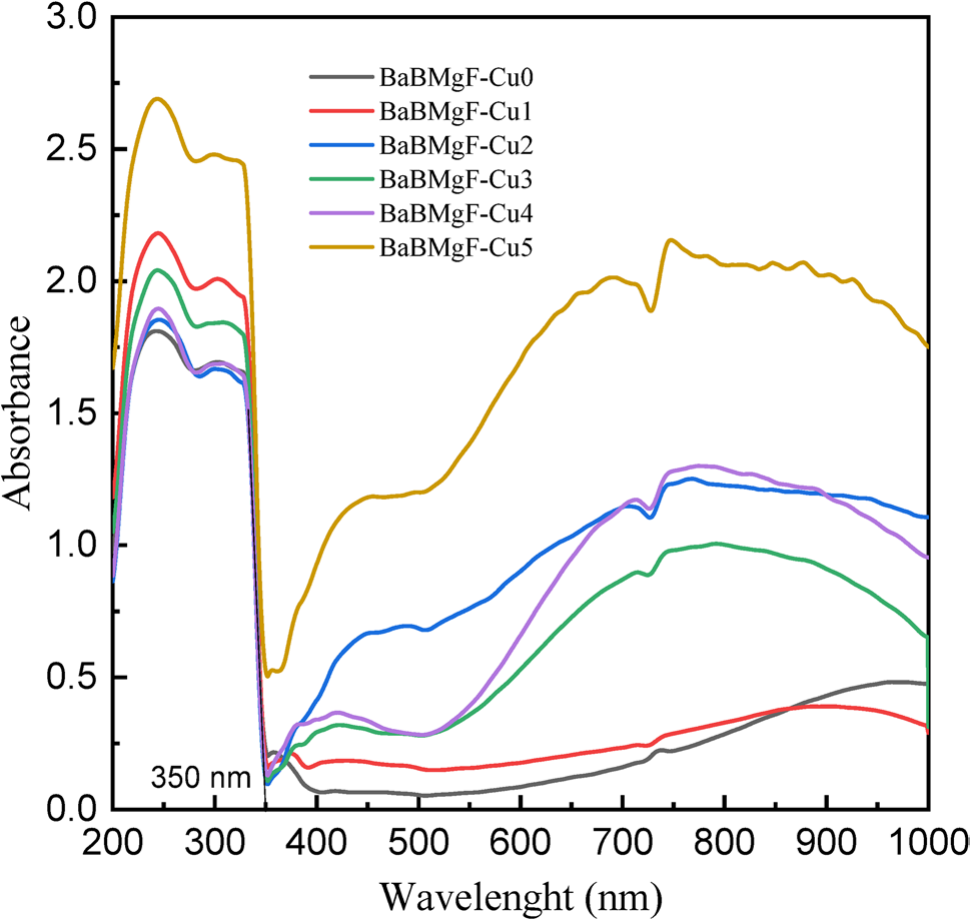
Optical absorption spectra for the studied glass samples.

The UV-visible absorption spectra of the glass samples were recorded in the wavelength range of 190–1000 nm, as shown in Fig. 7. The absorption spectra have two different UV absorption bands seen in their spectra: a low-intensity region around 310 nm and a high-intensity range at about 243 nm. Following a narrow absorption range, there appears an extensive band in the near-infrared spectrum between 550 and 1000 nm, centered at 765 nm for the doped glasses by CuO. The absorption intensity in the UV region (200–350 nm) shows marked enhancement with higher CuO concentrations. They clearly show extremely significant increases with increasing CuO content for the UV region between 200 and around 350 nm. The broad absorption band in the visible spectrum is consistent with established data on Cu^+ 2^ ions located at distorted coordination sites^40^.

**Table 2:** Optical parameters.

Optical parameters	Sample Name					
	Cu0	Cu1	Cu2	Cu3	Cu4	Cu5
UV cutoff Wavelength (nm)	350	350	350	350	350	350
Energy gap, Indirect allowed transition (eV)	3.48	3.48	3.48	3.48	3.48	3.48
Energy gap, Indirect forbidden transition (eV)	3.48	3.48	3.48	3.48	3.48	3.48
Energy gap, Direct forbidden (eV)	3.52	3.52	3.52	3.52	3.52	3.52
Energy gap, Direct allowed (eV)	3.6	3.6	3.6	3.6	3.6	3.6

**Fig. 8:**
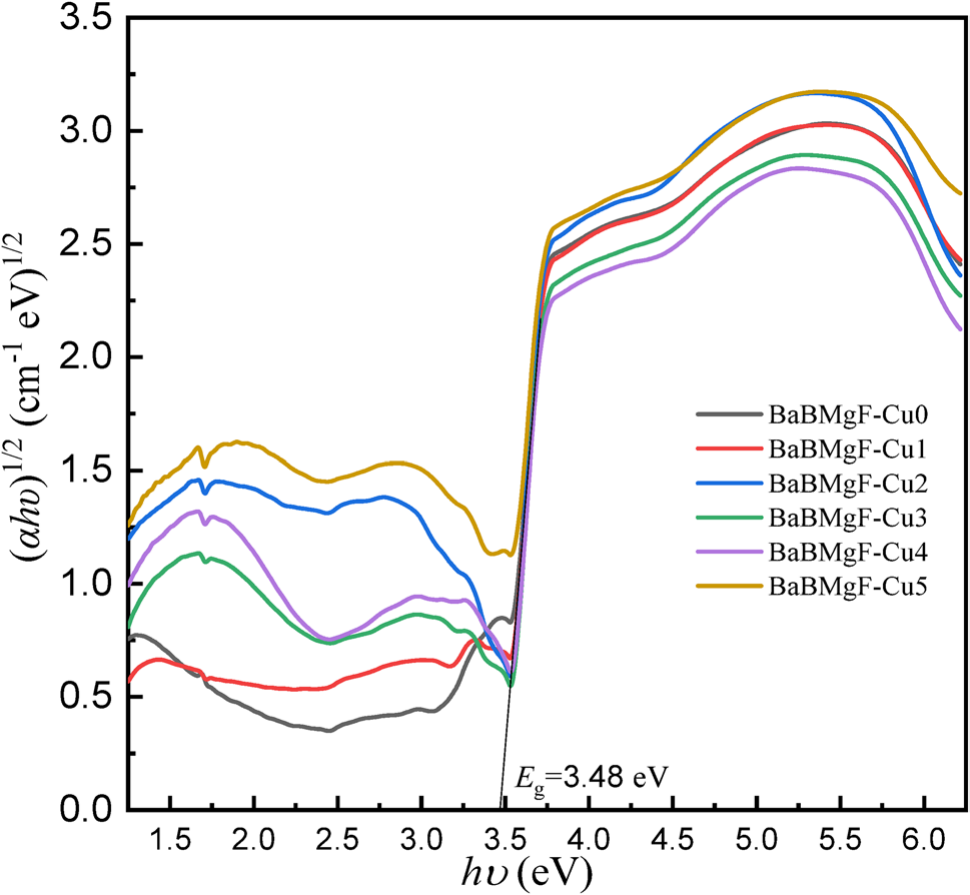
Optical energy gap for indirect allowed transition.

**Fig. 9:**
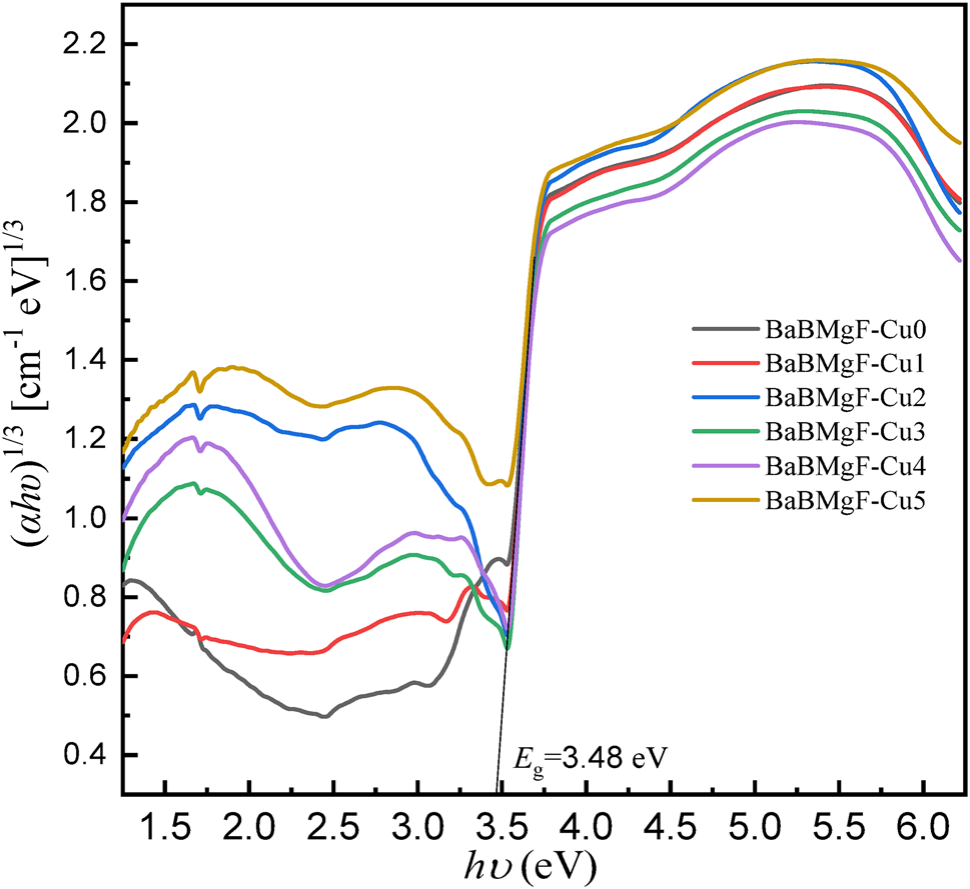
Optical energy gap for indirect forbidden transition.

**Fig. 10:**
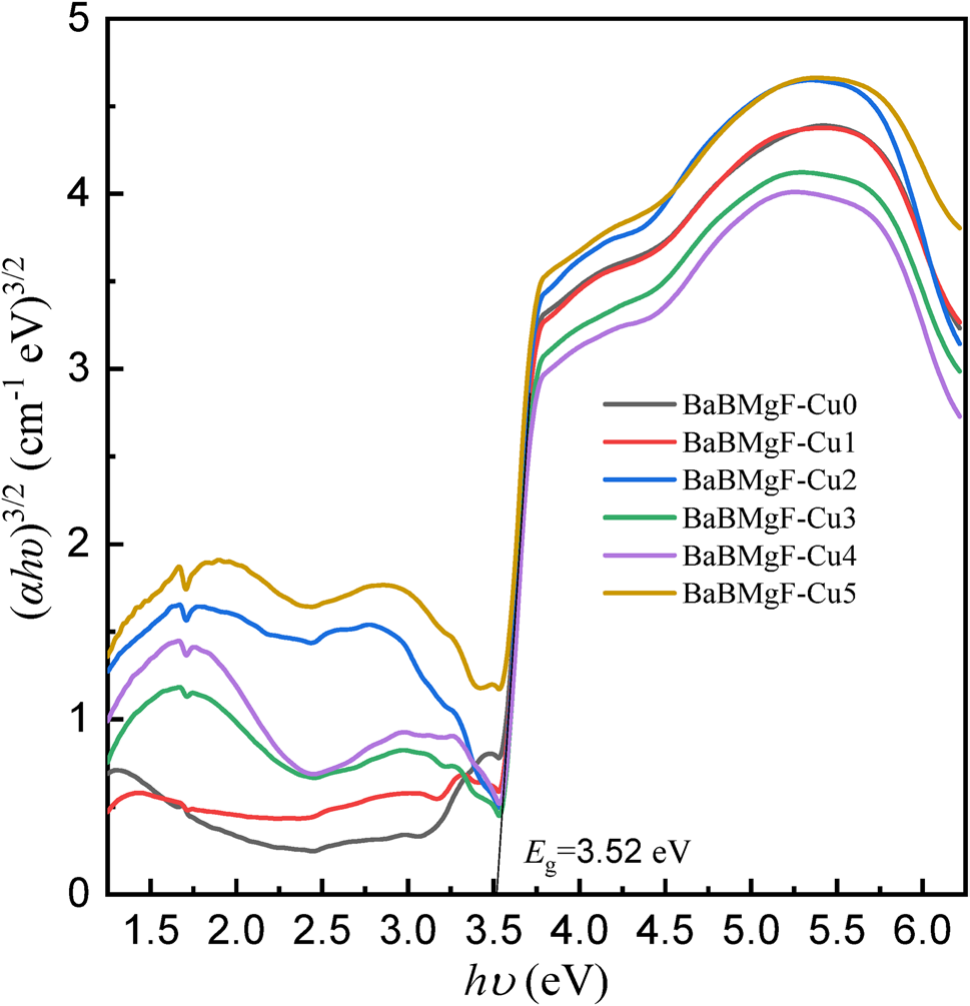
Optical energy gap for direct forbidden transition.

**Fig. 11:**
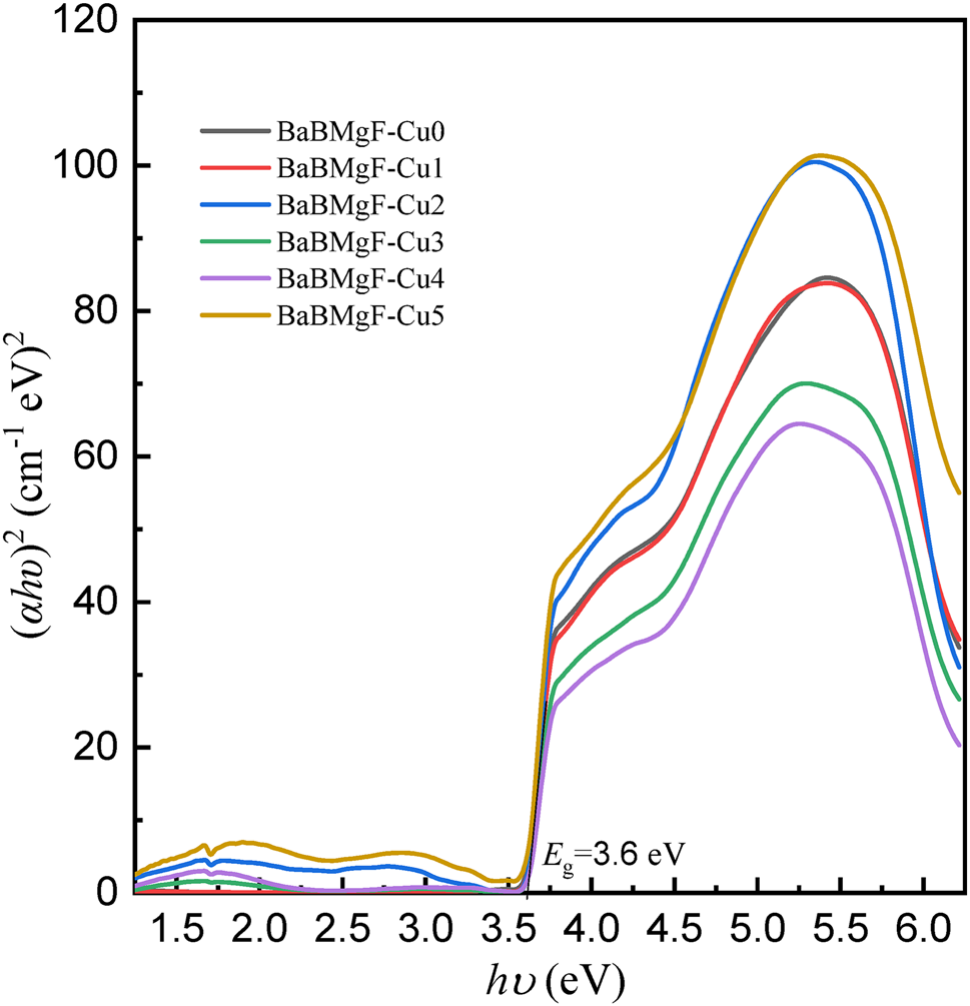
Optical energy gap for direct allowed transition.

Several optical parameters have been found using UV-Vis. Data and Table 2 provide further information. Figures 8 and 9 for indirect transmission and also Figs. 10 and 11 for direct transmission demonstrate the visually acquired results of the calculation of the optical energy gap values for both indirectly and directly allowed and also forbidden transitions using the Daff and Mott formula^41^.

For shielding materials to work at their best, improving their optical qualities is equally crucial. By choosing an ideal concentration, it is consequently feasible to enhance optical qualities while preserving adequate shield performance.

Dimitrov and Sakka (Davis and Mott 1971) claim that the optical band gap is connected to the linear refractive index (n) of the glasses, considering that the energy gap and the refractive index possess an inverse relationship.

The critical optical novelty arises from decoupling the bands for strong UV absorption and the band gap. This approach confirms that localized states for UV shielding are introduced by Cu²⁺ ions and that the wide bandwidth for transparency is unaltered. This selectivity is highly important for transparent shielding materials that can block UV but transmit visible light, unlike UV shielding glasses that turn dark and/or become opaque.

### Dielectric study

#### AC conductivity

**Fig. 12:**
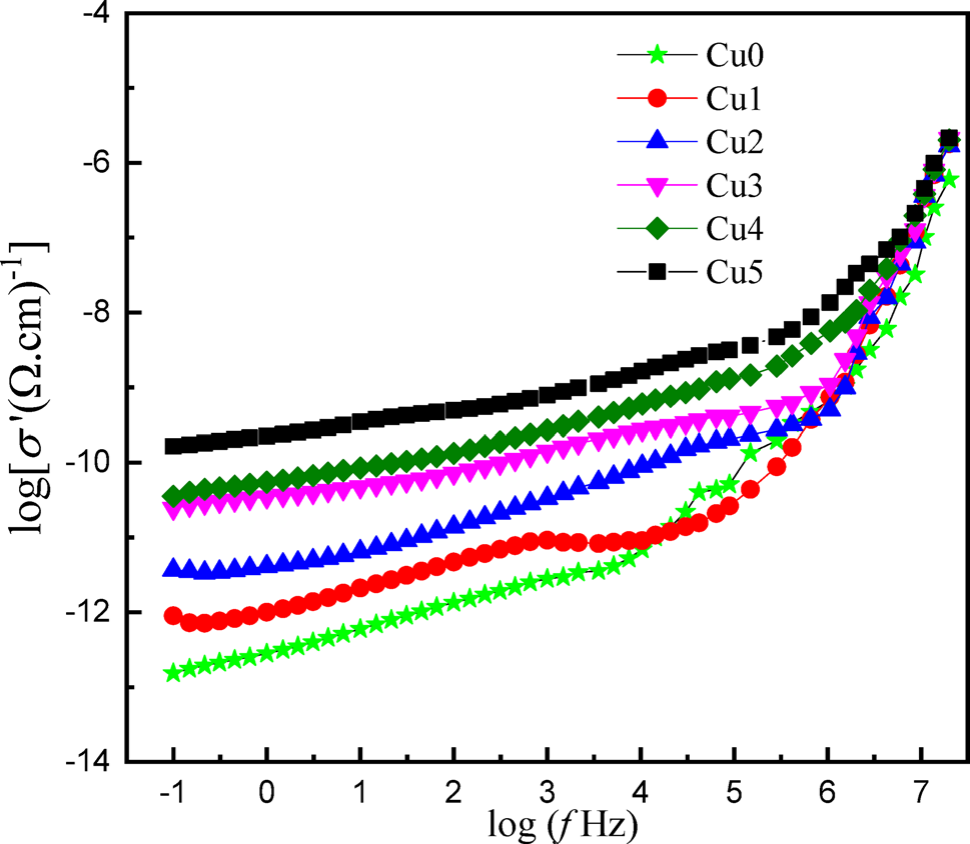
The variation of the real part of conductance with frequency of the *x*CuO-(50-*x*)B_2_O_3_−35BaO-15MgF_2_ Glasses.

Figure 12 represents the frequency-dependent electrical conductivity spectra of the *x*CuO-(50-*x*)B_2_O_3_−35BaO-15MgF_2_ glasses. The spectra exhibit systematic variations in charge transport mechanisms as a function of copper concentration. The logarithmic real part of conductivity log (σ’) versus frequency log (ω) at room temperature for the studied glasses, reveals characteristic glassy semiconductor behavior with three distinct regions according to Jonscher’s universal power law^42^.

$$s\left( w \right){\text{ }} = {s_{dc}} + {\text{ }}A{w^s}\;\;\;\;\left( {0{\text{ }} < {\text{ }}s{\text{ }} < {\text{ }}1} \right)$$

Where *σ*_dc_ is the dc conductivity, A is a temperature-dependent constant, (ω = 2πf) is the angular frequency of the applied field, and s is a power law exponent representing the degree of interaction between charge carrier (Cu^2+^, Ba^2+^, Mg^2+^).

At low frequencies (log f < 100 Hz), all compositions exhibit frequency-independent conductivity plateaus corresponding to DC conductivity (σ_dc_), which increases gradually from Cu0 (≈ 10^− 13^ (Ω·cm)^−1^) to Cu5 (≈ 10^− 10^ (Ω·cm)^−1^), indicating enhanced ionic conductivity with increasing copper content.

The intermediate frequency region (10^3^ < f < 10^5^ Hz) exhibits dispersive behavior where conductivity transitions from a frequency-independent to frequency-dependent region, with higher copper content samples (Cu4, Cu5) showing a sudden change of dispersion, indicating reduced activation barriers for charge carrier relaxation processes attributed to decreased spacing between ions and a higher density of defects in the samples. At high frequencies (f ≥ 10^5^ Hz), all samples demonstrate pronounced power-law conductivity increases following *σ*(ω) ∝ Aω^s 43^. Characteristic of correlated barrier hopping (CBH) between localized states, with remarkable convergence suggesting that microscopic charge dynamics based on Cu⁺/Cu²⁺ electron exchange, fluorine ion F^−^ vibrational frequencies, and nearest-neighbor coordination structures remain fundamentally similar despite macroscopic compositional differences.

**Fig. 13:**
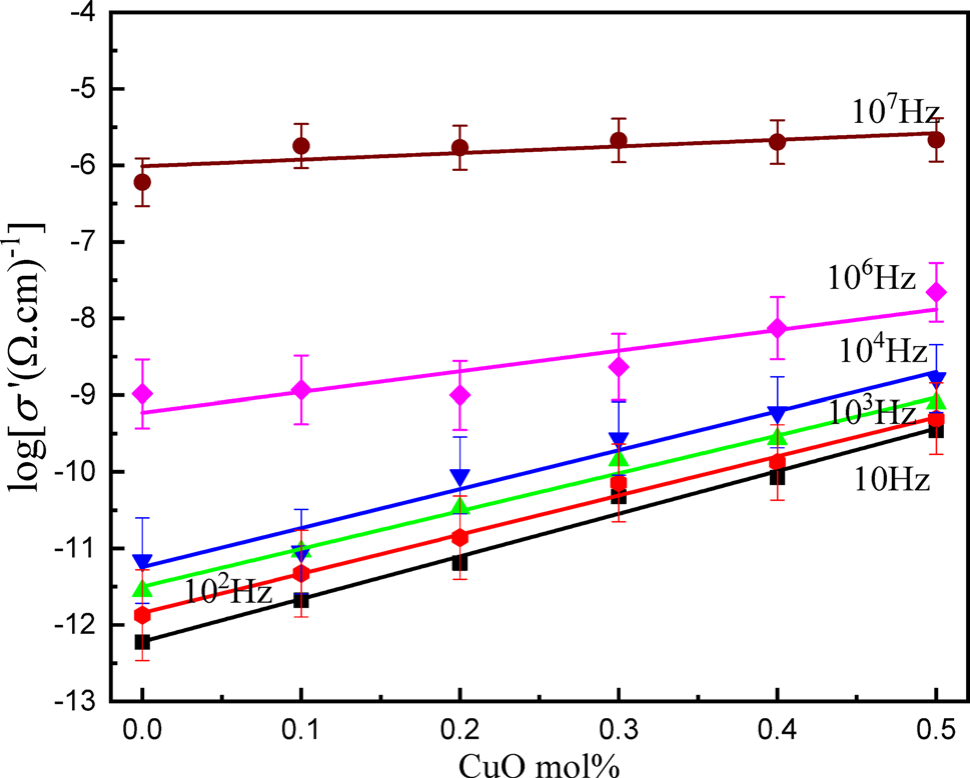
Dependence of conductivity as a function of CuO content in *x*CuO-(50-*x*)B_2_O_3_−35BaO-15MgF_2_ Glasses.

Figure 13 represents the composition dependence of conductivity at different frequencies for the studied glasses. DC and AC conductivity are significantly enhanced with increasing CuO content. There is a linear increase in both types of conductivity (DC and AC) with CuO content at different frequency values. The increase in the DC conductivity from − 11.87 to −9.3 (Ω.cm)^[- 1^ at 100 Hz can be attributed to an increase in the number of main charge carriers and also the formation of conduction pathways through the glass network. At the high frequency region, AC conductivity increases with increasing frequency, following the universal dielectric response law, tends to (−5.66 (Ω.cm)^[-1^) at 10^7^ Hz for Cu5. Generally, the conductivity is significantly influenced by CuO concentration.

#### Dielectric properties

The complex permittivity *ε* ^*^ is used to describe the dielectric properties of materials, which is based on the real dielectric constant *ε* and the imaginary dielectric loss *ε*. The complex permittivity is evaluated from the following relation,

$${e^*} = {e^{'{\text{ }} + {\text{ }}i}}{e^{\prime \prime }}$$

**Fig. 14:**
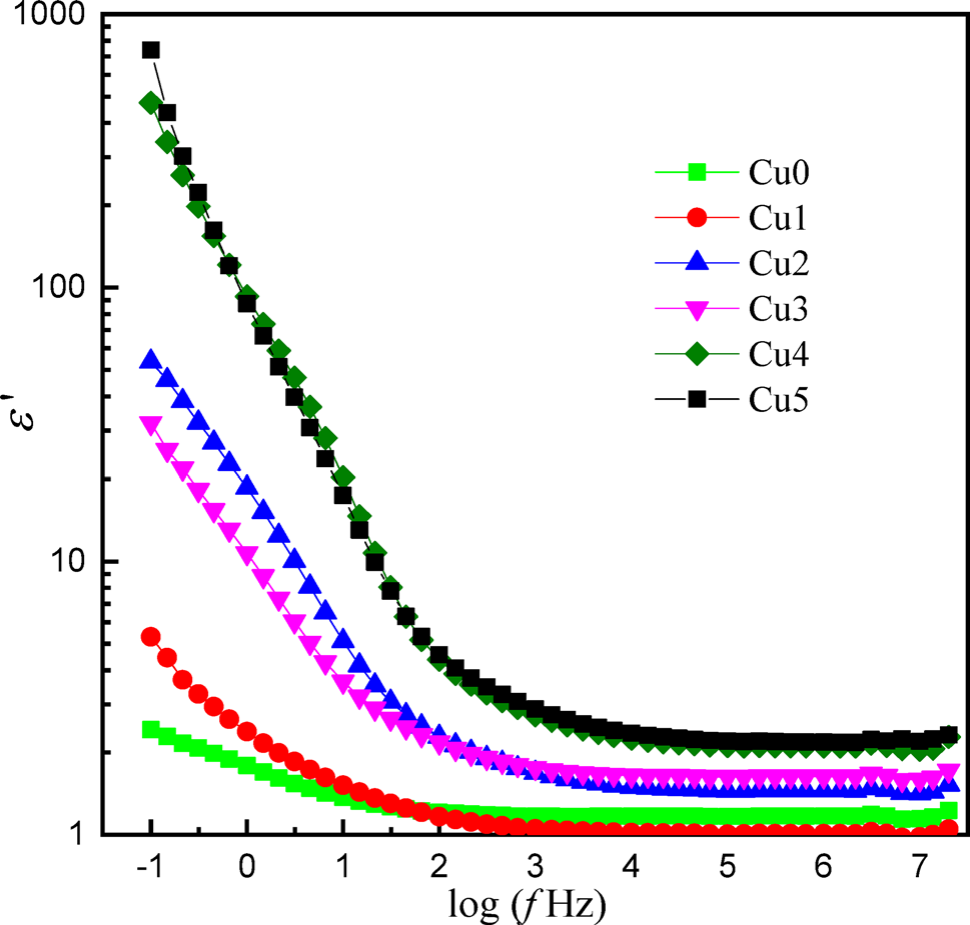
The variation of the real part of the dielectric constant with frequency of the *x*CuO-(50-*x*)B_2_O_3_−35BaO-15MgF_2_ Glasses.

**Fig. 15:**
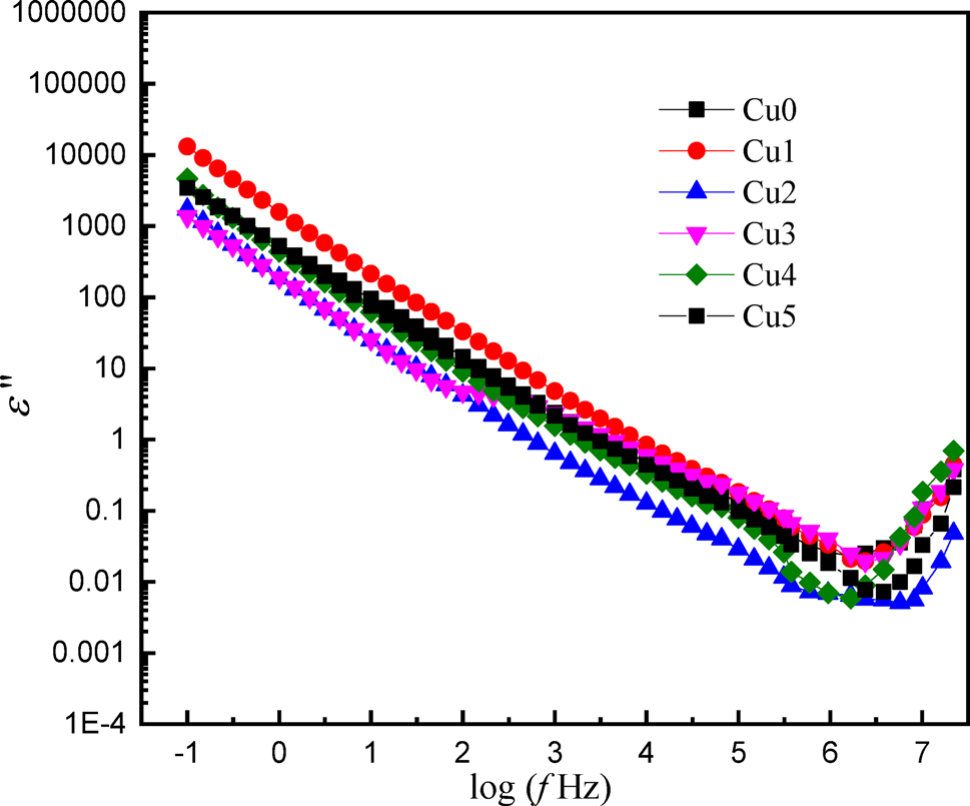
The variation of the imaginary part of the dielectric constant with frequency of the *x*CuO-(50-*x*)B_2_O_3_−35BaO-15MgF_2_ Glasses.

The frequency-dependent behavior of the real part (*ε*′) and imaginary part (*ε*′′) dielectric constant was examined for the studied glass doped with CuO content. Figures (14 and 15) show the dependence of *ε*′ and *ε*′′ on frequency for the (0 −0.5 CuO) samples; both parameters decreased with increasing frequency. The behavior of *ε*′ can be attributed to dipolar and interfacial polarization mechanisms, which result from the orientation of permanent dipoles in the electric field direction, which prevent the passage of the mobile ion (Cu^2+^, Ba^2+^, Mg^2+^) through the external circuit^44^.

Whereas *ε*′′ is obtained from both dc conduction and the dielectric polarization. At very low frequency, all samples exhibit high dielectric loss values, particularly for sample Cu1, which reaches 10^4^, which may be due to the presence of a structural defect. This increase can be explained by ionic conductivity contribution and Maxwell-Wagner-Sillars interfacial polarization phenomena, by accumulation of charge carriers at (Cu^2+^, Ba^2+^, Mg^2+^) the boundaries between the different structure units in the network^45^. With increasing frequency ε’’ decrease for all samples is observed, which is related to the contribution of ionic conductivity. All samples have a slight increase at higher frequencies, higher than 10^6^ Hz, which may be due to specific dielectric relaxation processes or resonance effect within the matrix components. This behavior agrees with the existence of charges that can respond to high frequency field, due to dipolar or orientation losses.

In addition, the sample displays an exceptionally high dielectric constant value, sample Cu5 reaching a value approaching 700, while sample-free CuO has the lowest value, approximately 2.5. The present result indicates that the studied glass capability for utilization in technological applications related to energy storage and conversion systems.

The most prominent electric innovation involves the creation of a semiconducting-like state with very low doping (≤ 0.5 mol% CuO). The dramatic three-order-of-magnitude enhancement of DC conductivity and the huge low-frequency dielectric constant (ε’ ∼ 700) occur due to Cu⁺/Cu²⁺ hop-polarization and interfacial contributions. Notably, it has been evidenced that low concentrations of the transition metal ion can substantially influence the electric properties of an insulating fluoroborate glass to produce a multifunctional phase amenable to both capacitive energy storage and solid-state device applications that require optical transmission.

### Shielding effectiveness

**Fig. 16:**
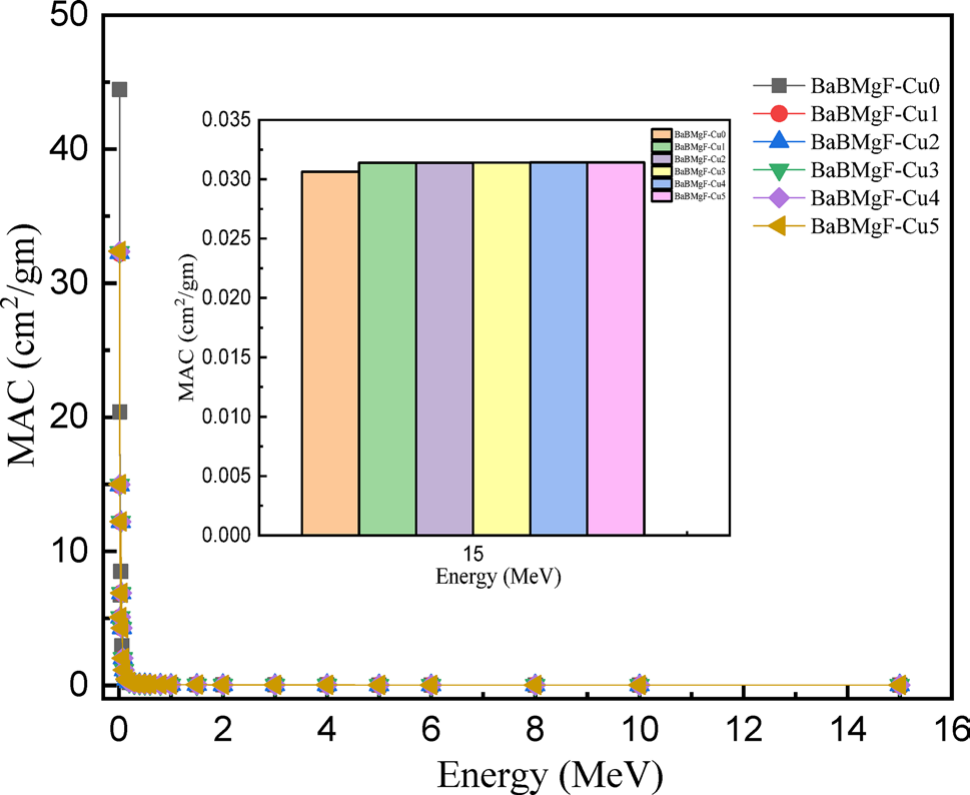
The MAC values versus incoming photon energy for all studied glasses.

**Fig. 17:**
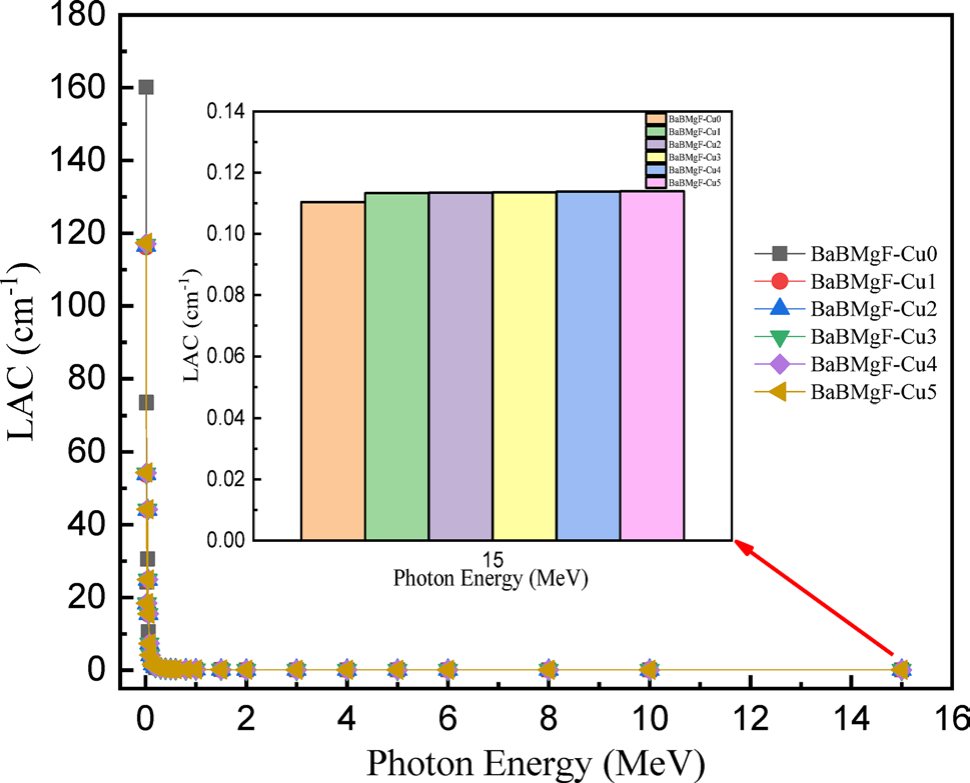
The LAC values versus incoming photon energy for all studied glasses.

The prepared samples’ shielding properties are examined using a new piece of software called Phy-X/PSD. For all prepared samples, the linear and mass attenuation coefficients are shown vs. photon energy in the range of 0.015–15 MeV in Figs. 16 and 17. Because of the photoelectric interaction process, it is found that both MAC and LAC exhibit the largest value at lower energy (E = 0.015 MeV), and that their values fall with increased photon energy, reaching the lowest value at higher energy (E = 15 MeV). Compton scattering accounts for the majority of this drop^46,47^.

According to the MAC and LAC attenuation factor data (Figs. 16 and 17), substituting CuO for the glass matrix with different concentrations results in moderate values in both attenuation factors (MAC and LAC). This can lead to changes in shielding efficiency because of the mixed alkali effect. Of the varieties of glass under investigation that were previously disclosed, glass sample Cu 5 showed the highest MAC and LAC values.

**Fig. 18:**
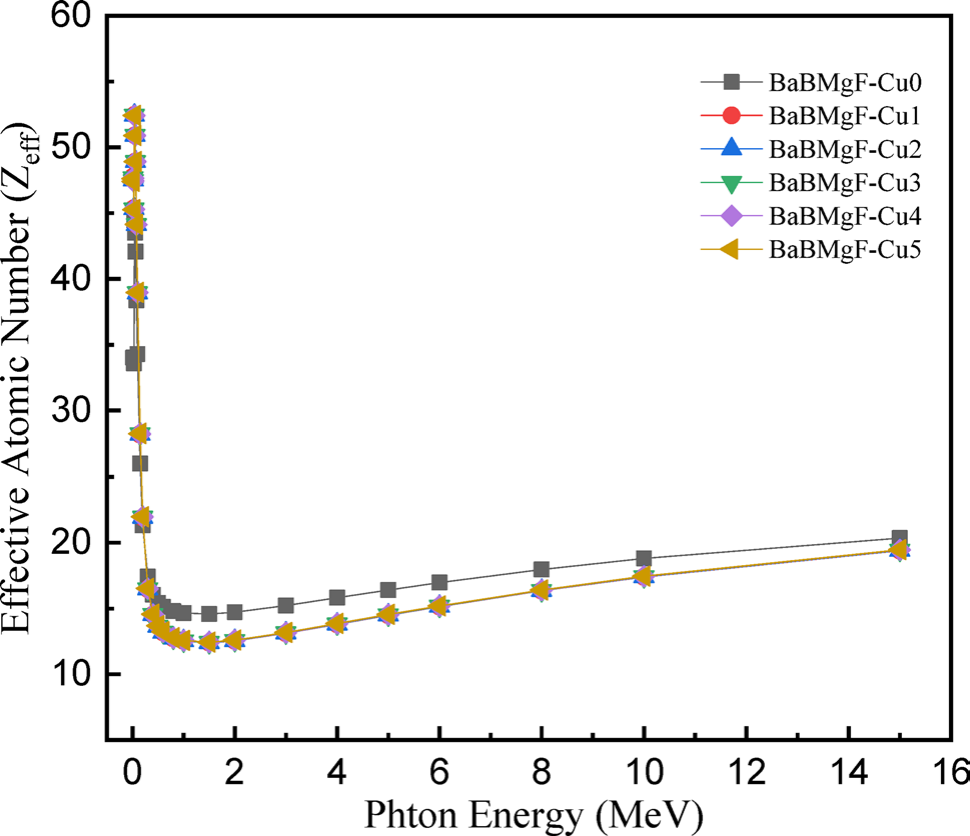
Zeff values versus incoming photon energy for all studied glasses.

Other crucial radiation physics parameters that are necessary for shielding studies include the effective atomic number (commonly represented as Zeff) and mean free path (typically represented as MFP). In order to assess the shielding effectiveness against energetic photons, the Zeff calculation is essential Fig. 18. It functions as a gauge of the electronic cloud engaged in the interaction process between photons and provides preliminary information about the attenuation capacity of the used shield^48^.

**Fig. 19:**
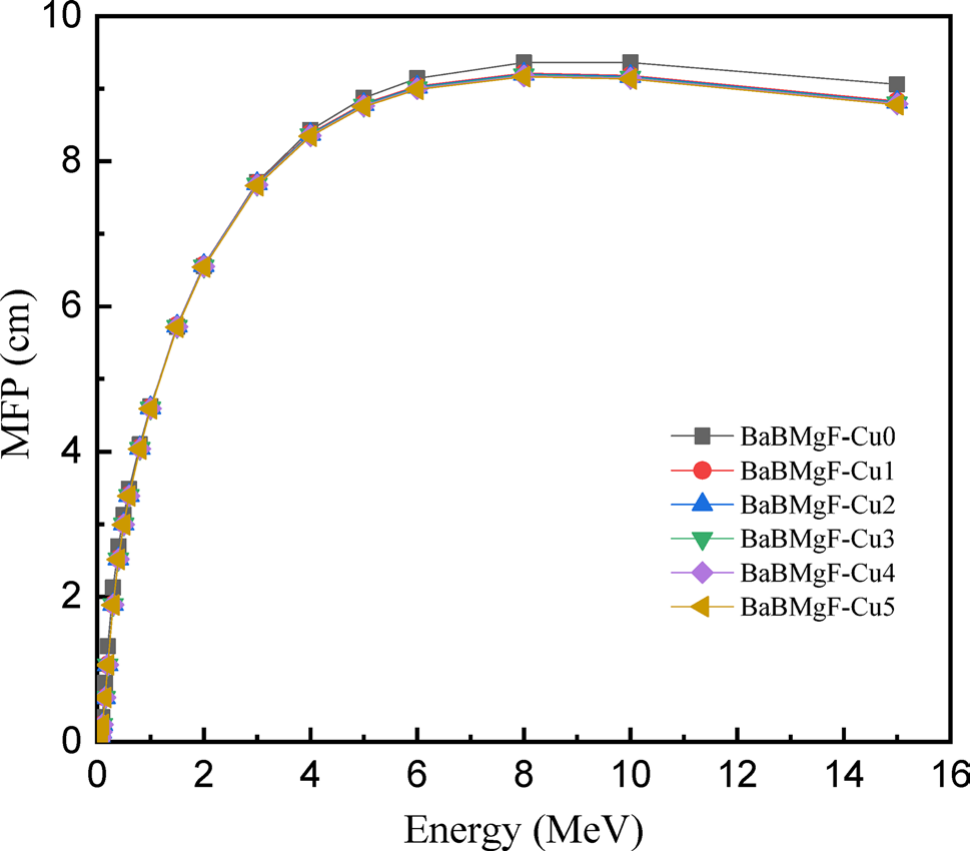
MFP values versus incoming photon energy for all studied glasses.

The mean free path, or MFP, is the average distance that photons in a substance travel before encountering another interaction. It plays a crucial role in comparative research by assessing the impact of composition and doping alterations on this interaction length. All produced glass samples’ MFPs are plotted as a function of energy in Fig. 19. As photon energy rises, the MFP is observed to grow, reaching greater levels at 15 MeV. The shielding capacity would be enhanced by compositional changes that lower MFP at higher energy. With the lowest MFP value of all the prepared samples, Cu5 is a viable contender to serve as a shielding material, although all samples have the same effect.

**Fig. 20:**
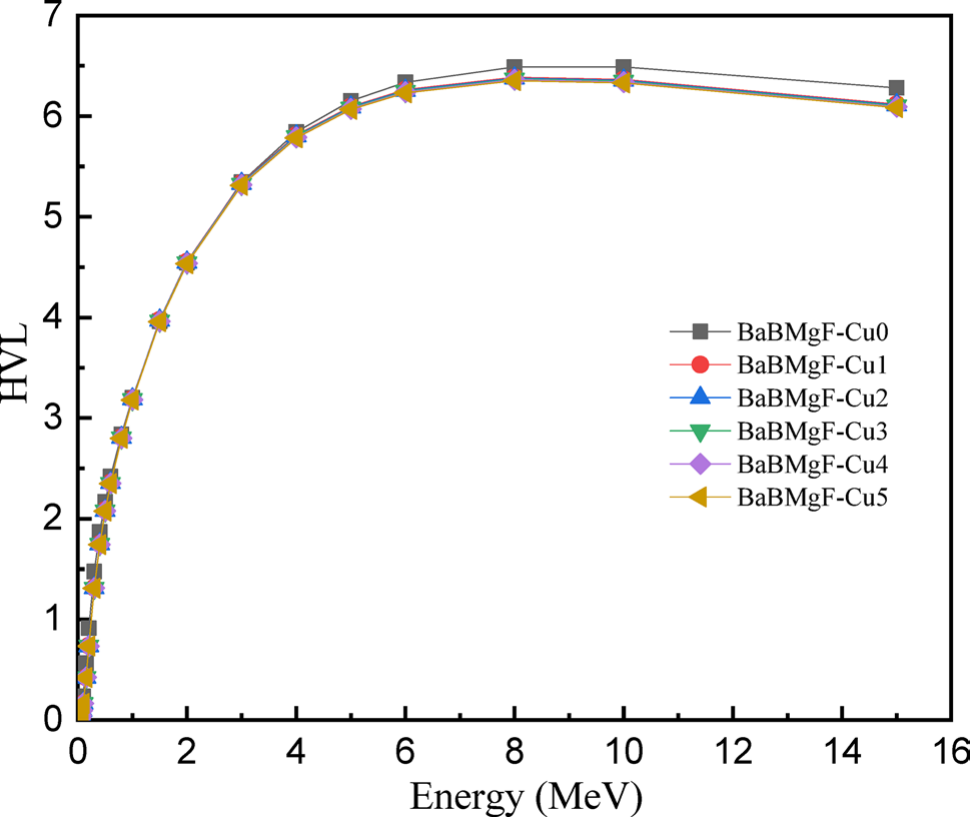
The HVL values versus incoming photon energy for all fabricated samples.

**Fig. 21:**
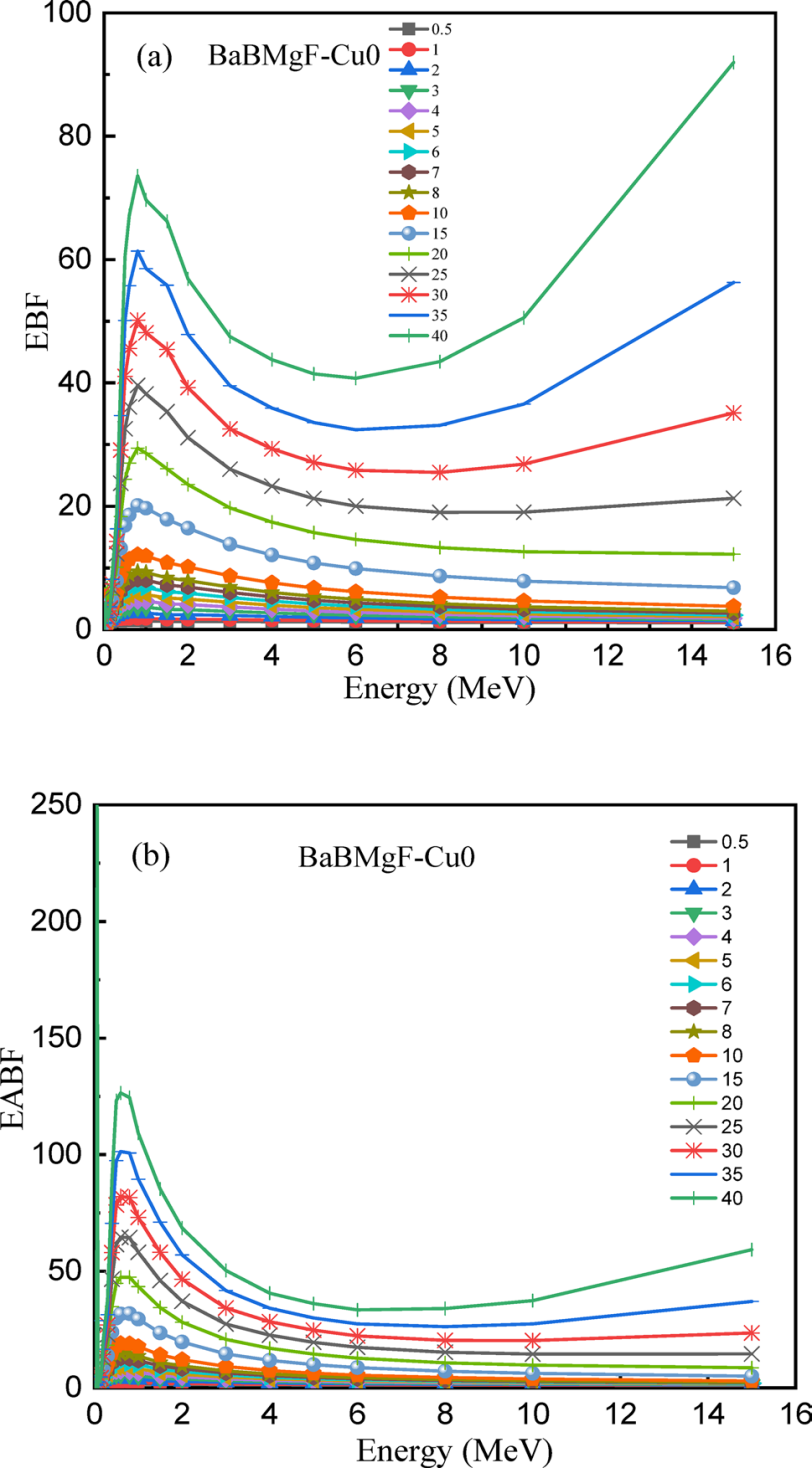
The energy build factor (EBF) (**a**) and the energy absorption build factor (EABF) (**b**) values versus the change of photon energy for the Cu0 sample. The HVL values versus incoming photon energy for all fabricated samples.

**Fig. 22:**
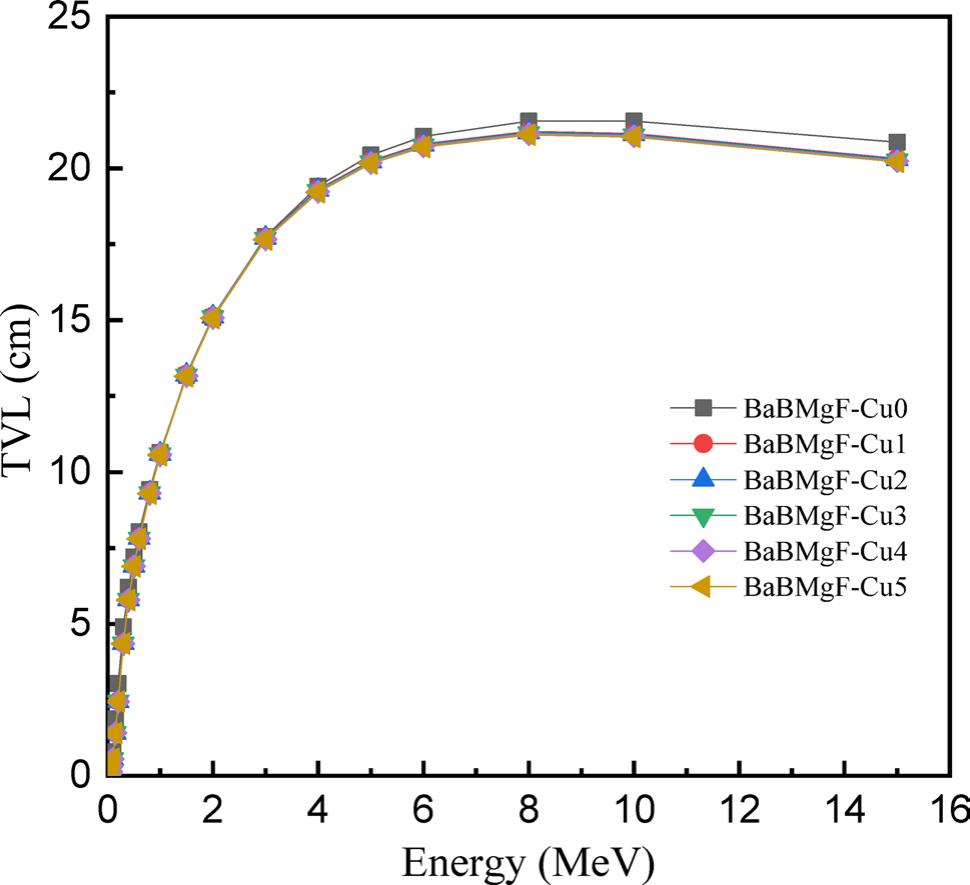
The TVL values versus incoming photon energy for all studied samples.

As photon energy rises, the MFP is observed to grow, reaching greater levels at 15 MeV. The shielding capacity would be enhanced by compositional changes that lower MFP at higher energy. With the lowest MFP value of all the prepared samples, Cu5 is a viable contender to serve as a shielding material. The factors of half and tenth value layers are low for glass materials that have effective radiation shielding characteristics. For every research, the half and tenth-value layers are displayed as a function of energy in Figs. 21 and 22 between 0.015 and 15 MeV in terms of samples. As energy increases, both the HVL and TVL values progressively grow; the highest values were recorded at 15 MeV.

TVL (Fig. 20) has higher quantitative values at the same energy level as HVL. As seen in Figs. 16 and 17, the inclusion of CuO into the glass matrix resulted in a decrease in the half and tenth value layers of the research samples. Out of all the samples examined, the one with the lowest HVL and TVL is Cu0. It is capable of shielding enough photons to lower the radiation intensity and maintain a level.

Buildup factors [EPF & EAPF] are the best shielding characteristics that inform us about the efficacy of shielding materials. In order to understand the contributions of interacting and non-interacting photons during absorption and to clarify how materials interact with incoming gamma rays, it is imperative to understand the accumulation components. Exposure buildup factors (EBF) and energy absorption buildup factors (EABF) are the two categories of buildup factors.

**Fig. 23:**
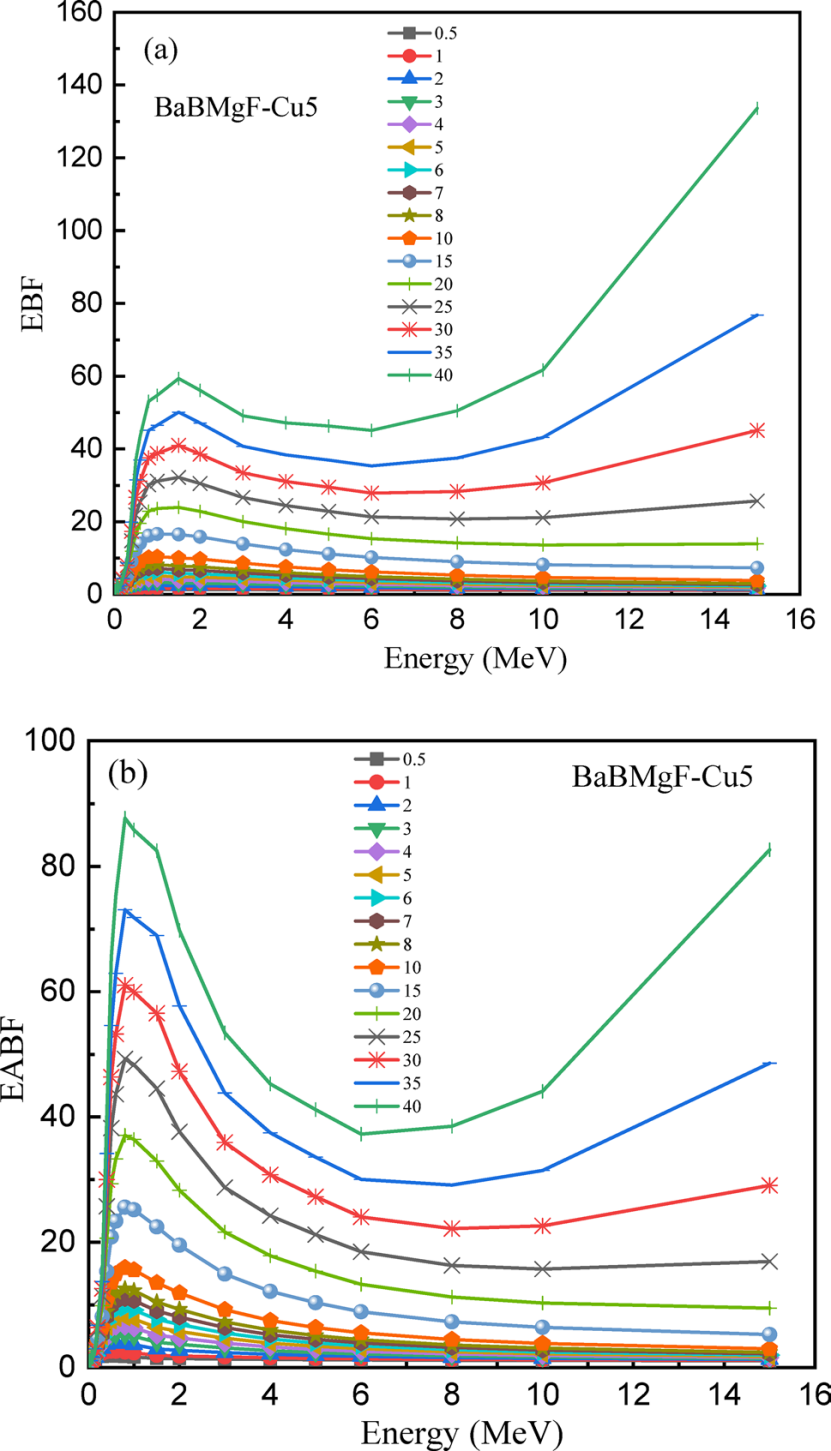
The energy build factor (EBF) (**a**) and the energy absorption build factor (EABF) (**b**) values versus the change of photon energy for the Cu5 sample.

The EBF and EABF factors for the chosen samples, Cu0 and Cu5, as a function of energy are shown in Figs. 22 and 23, respectively. At moderate photon energy, the highest values are seen to occur. Since the maximum accumulation happens away from the extremes, this emphasizes how crucial it is to take buildup effects into account when evaluating shielding over a range of gamma-ray energies. The intricate gamma ray attenuation behavior of these glass materials is revealed by the EBF and EABF profiles. When the incident photon energy is increased, it is seen that both EBF and EABF have small values at lower energy levels. These values subsequently increase to achieve maximum values at medium energy levels, and then they start to drop as photon energy is sustained.

These shielding experiments exhibit a new type of synergy, namely, the cumulative effect of adding increasingly smaller amounts of CuO within the high Z matrix of BaO/B₂O₃/MgF₂ translates directly into a measurable improvement of gamma ray attenuation coefficients. These findings not only validate our prediction of improving shielding by adding dopants but, by extension, define CFBB not only as a lead substitute, but as a multi-component active shielding platform. In essence, we can now mutually tailor optical, dielectric, as well as shielding properties.

## Conclusion

This systematic research has successfully accomplished its aim of tailoring and assessment of the multiple functionality properties for CuO-doped fluorobarioborate glasses. The major findings satisfy the research aims based on the following:

- The analysis of XRD and FTIR spectra showed that a CuO concentration of less than or equal to 0.3 mol% is doped without compromising the borate network, with a stable BO₃/BO₄ ratio, N₄ ≈ 0.59, while beyond 0.5 mol%, sol.
- The UV-Vis spectroscopy indicated that CuO was an effectiveUV absorber because of localized Cu²⁺ transitions revealed by enhanced edge features in the 200–350 nm range, whereas the fundamental optical band gap of ~ 3.5–3.6.5.6 eV was unchanged.
- Analysis of the AC conductivity and dielectric studies showed the effect of the addition of CuO to the glass, which changed it from an insulator to a semiconductor material by increasing the DC conductivity by three orders of magnitude through Correlated Barrier Hopping (CBH) polaronic conduction and also caused the value of the permittivity to increase to 700 at low frequencies by Maxwell-Wagner-Sillars polarization.
- Phy-X/PSD showed that there was an improvement in the shielding properties (LAC, MAC, Zeff) depending on the concentration of CuO used. The glass with high concentration of copper oxide (Cu5) showed the lowest HVL, which asserts that it has better attenuation properties, especially at lower energies.

The aim of this study has been successfully accomplished by designing a single glass that combines an efficient radiation shielding factor, controlled UV absorption, stable dielectric properties, and mechanical strength. The unique properties required in CuO-doped CFBB glasses make them an efficient and multi-functional suitable alternative to traditional poisonous lead shielding glasses used in advanced technological applications.

## Data Availability

All data generated or analyzed during this study are included in this published article.
